# Paternal Deprivation and Female Biparental Family Rearing Induce Dendritic and Synaptic Changes in *Octodon degus*: I. Medial Prefrontal Cortex

**DOI:** 10.3389/fnsyn.2020.00038

**Published:** 2020-09-04

**Authors:** Tony de Schultz, Joerg Bock, Katharina Braun

**Affiliations:** ^1^Department of Zoology, Developmental Neurobiology, Institute of Biology, Otto von Guericke University Magdeburg, Magdeburg, Germany; ^2^PG “Epigenetics and Structural Plasticity,” Institute of Biology, Otto von Guericke, University Magdeburg, Magdeburg, Germany; ^3^Center for Behavioral Brain Sciences, Magdeburg, Germany

**Keywords:** dendritic spines, prefrontal cortex, paternal deprivation, family structure, sex difference, pyramidal neuron

## Abstract

In most mammalian species parent-offspring interactions during early life periods primarily comprise social contacts with the mother, whereas the role of males in parental care is one of the most overlooked and understudied topics. The present study addressed the hypothesis that the complete deprivation of paternal care delays or permanently retards synaptic connectivity in the brain, particularly in the medial prefrontal cortex (mPFC) of the offspring in a sex-specific manner. Another aim of this study was to address the question whether and in which way replacing the father with a female caregiver (in our experiments the “aunt”) can “buffer” the detrimental effects of paternal deprivation on neuronal development. The comparison of: (a) single mother rearing; (b) biparental rearing by father and mother; and (c) biparental rearing by two female caregivers revealed that: (i) paternal care represents a critical environmental factor for synaptic and dendritic development of pyramidal neurons in the vmPFC of their offspring; (ii) a second female caregiver (“aunt”) does not “buffer” the neuronal consequences of paternal deprivation; and that (iii) neuronal development in the vmPFC is differentially affected in male and female offspring in response to different family constellations.

## Introduction

The vast majority of studies emphasize the important role of the mother in supporting infant growth and survival and facilitating cognitive as well social-emotional development (Meaney, 2001; Zhang et al., 2013; Bock et al., 2014; Maccari et al., 2014; Glynn and Baram, 2019). In contrast, the father’s contribution to infant development has been less studied. This is surprising since in humans as well as in several other bi-parental mammalian species the father represents a major source of emotional and social interactions (Feldman et al., 2019). Recent studies comparing maternal and paternal behavior in humans showed that fathers provide the same level of sensitivity as mothers during interactions with their children and make a unique contribution to the child’s social development and emotion regulation, particularly to the child’s later capacity to function adaptively within the social milieu (Feldman, 2016; Kohl et al., 2017, 2018; Abraham and Feldman, 2018). Absence of a paternal caregiver influences the socialization and increases the probability of drug and alcohol abuse, mental illness, poor educational performance and criminal activity of affected children (Franz et al., 1999; Baskerville, 2002; O’Neill, 2002; Erhard and Janig, 2003; Kindler and Grossmann, 2004; Garfield and Isacco, 2006). Also, a variety of clinical studies provide evidence that adolescents living in single-mother homes develop higher levels of delinquency than those raised in dual-parent households (Juby and Farrington, 2001; Demuth and Brown, 2004) and are at greater risk for incarceration (Harper and McLanahan, 2004).

So far there are no systematic analyses of brain functional changes in father-deprived children, which underlines the importance of research in animal models, which—besides, to use functional imaging techniques—also allow to “zoom in” to the microscopic and molecular/epigenetic level to study the impact of paternal care on neuronal and synaptic development. An increasing number of experimental approaches in various biparental rodent species revealed the important role of fathers and bi-parental care in shaping functional neuronal networks in the brain and the associated socioemotional development of their offspring (Braun and Champagne, 2014; Bales and Saltzman, 2016; Saltzman et al., 2017; Feldman et al., 2019; Pohl et al., 2019). These studies compared the developmental trajectories of offspring from biparental and single-mother families, whereas studies investigating possible influences of an additional (male or female) caregiver are rare.

We have introduced the trumpet-tailed rat *Octodon degus* as an animal model to study developmental processes underlying human psychopathologies in the context of the DOHaD (Developmental Origins of Health and Disease; Hanson and Gluckman, 2014; Heindel et al., 2015). This species is characterized by biparental care that is associated with complex familial structures and an intense social bond between young degus and their parents (Fuchs et al., 2010; Colonnello et al., 2011). Behavioral studies in degus have shown that the father is actively engaged in parent-offspring interactions (Wilson, 1982), including huddling, licking and grooming, and play behavior (Helmeke et al., 2009; Pinkernelle et al., 2009). During the first 3 weeks of life paternal care comprises about 40% of total parent-offspring interactions (Helmeke et al., 2009; Pinkernelle et al., 2009) and it was claimed that degu fathers play a major role as “regulator” of the offspring’s behavioral development (Wilson, 1982). Importantly, after the removal of the father single degu mothers do not compensate for the lack of paternal care by intensifying maternal activities (Helmeke et al., 2009), which creates a socio-emotionally deprived environment in fatherless families. Degus share the principal brain anatomy with common laboratory rodents (Wright and Kern, 1992; Kumazawa-Manita et al., 2013, 2018) and they display superior cognitive functions, such as learning to use a tool to retrieve food (Okanoya et al., 2008). Similar to human babies (De Casper and Fifer, 1980; Fifer and Moon, 1994), newborn degus learn to recognize and to respond to their mothers’ vocalizations within the first days of life (Poeggel and Braun, 1996; Braun and Scheich, 1997; Braun and Poeggel, 2001), and also similar to humans this vocal communication is important for the establishment and maintenance of the emotional attachment to the parents.

Various experimental studies analyzing the development of sensory and motor systems revealed that the developing brain is “experience-expectant” during circumscribed critical periods and that the establishment and refinement of sensory and emotional brain pathways requires an “enriched” stimulating environment during these life periods (Bryan and Riesen, 1989; Rosenzweig and Bennett, 1996; Bock and Braun, 1999a,b; Poeggel et al., 2003; Markham and Greenough, 2004; Bock et al., 2014). Such an enriched socio-emotional environment is normally provided by an adequate and structured parental care. While biparental care can be envisioned as an “enriched” environment for the offspring, being raised by a single caregiver represents an “impoverished” environment, a family setting which in animal studies can be experimentally applied to assess the impact of paternal care on the development of his offspring. The behavioral pathologies observed in father-deprived individuals likely arise from a socio-emotionally impoverished family setting resulting in functional deficits within specific brain circuits. Indeed, the development and functional maturation of brain regions such as the orbitofrontal cortex (OFC) and the anterior cingulate cortex (ACd) are particularly sensitive towards paternal input as revealed in previous studies (Ovtscharoff et al., 2006; Helmeke et al., 2009; Braun et al., 2011, 2013; Seidel et al., 2011).

One aim of the present study was to address the hypothesis that paternal deprivation delays or permanently retards synaptic connectivity in the brain, particularly in the medial prefrontal cortex (mPFC) of the offspring. Most studies consider paternal absence synonymous with living in a single-parent, non-intact, or mother-headed family, and accordingly, the majority of experimental paternal deprivation studies applied a paradigm in which the father is removed from the family at the birth of his offspring. However, in “real” life an absent father can be replaced by other male or female caregivers, which are integrated into the family setting. Hence, the second aim of this study was to address the question whether the father can be replaced by another female caregiver (in our experiments the “aunt”) who may “buffer” the detrimental effects of paternal deprivation on brain development. Since so far, the majority of animal studies have focused on the consequences of paternal deprivation in male offspring, the third aim of this study was to compare the impact of paternal deprivation on neuronal development in male and female siblings.

## Materials and Methods

### Animals

The degus used in this study were bred in our colony at the Institute of Biology, Otto von Guericke University Magdeburg. They were housed in wire cages (l/w/h: 50 cm/42 cm/67 cm) in temperature (22°C) and humidity (55%) controlled rooms under a 12/12 h light/dark cycle. Freshwater and rodent diet pellets were accessible *ad libitum*, vegetables were added occasionally. All experiments were performed following the European Community’s Council Directive and according to the German guidelines for the care and use of animals in laboratory research. The experimental protocols were approved by the ethics committee of Saxony-Anhalt.

### Experimental Groups

Four weeks after mating a male and female breeding pair the sister of the female (aunt) was introduced to the breeding pair and housed in the same cage. On the day of delivery (postnatal day, PND, 0) the families were assigned to three experimental groups:

Control family (+F group = male and female biparental): after delivery of the pups the aunt was removed from the family, resulting in a bi-parental family in which the pups were raised by the father and mother (N_offspring_ = 7 males, 5 females, each from different families to prevent litter effects). The data presented for this group include a total of 28 neurons for males and a total of 20 neurons for females. The data presented for this group include a total of 28 neurons for males and a total of 20 neurons for females.

Single mother family (−F group = father-deprived): on the day of delivery the father and the aunt were removed from the home cage, resulting in a single-parent family in which the pups were raised by the mother only (N_offspring_ = 6 males, 7 females, each from different families to prevent litter effects). The data presented for this group include a total of 24 neurons for males and a total of 28 neurons for females.

Mother-Aunt family (MA group = female biparental): on the day of delivery the father was removed from the family, resulting in a bi-parental family in which the pups were raised by the mother and her sister (aunt; N_offspring_ = 6 males, 6 females, each from different families to prevent litter effects). The data presented for this group include a total of 24 neurons for males and also a total of 24 neurons for females. All families were group-housed in their home cage until postnatal day 45 (puberty).

### Quantitative Neuromorphology

**Figure 1:**
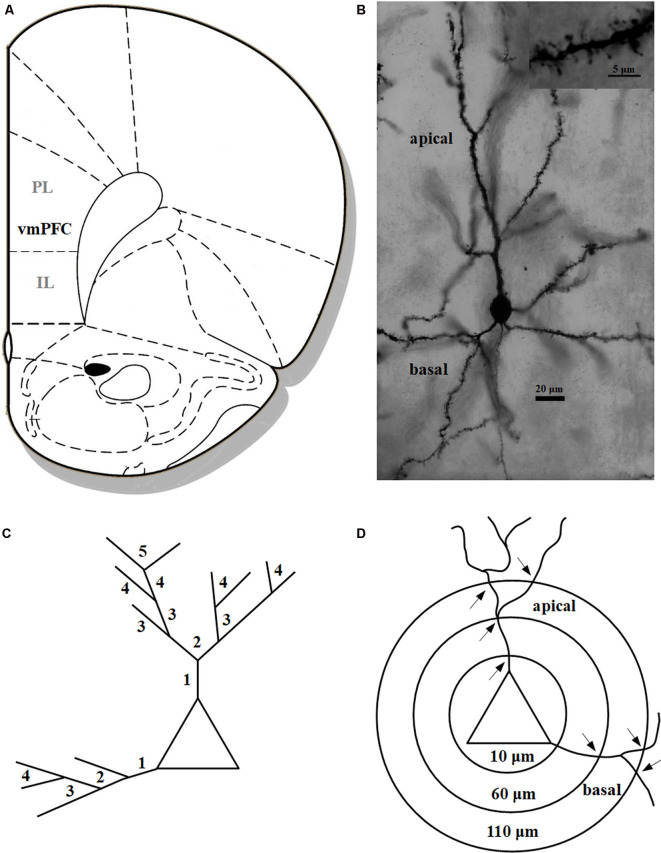
**(A)** Schematic illustration indicating the vmPFC in the Degu cortex comprising the infralimbic (IL) and prelimbic (PL) cortex (modified after Paxinos and Watson, 2004); **(B)** photomicrograph of a pyramidal neuron in the vmPFC; inset shows a dendritic segment with dendritic spines; **(C)** schematic illustration of dendritic branching with numbers indicating individual branch orders (“natural” dendritic segments); **(D)** schematic illustration of Sholl analysis with concentric spheres around the soma and respective radii in μm; arrows indicate intersections on apical and basal dendrites (“artificial” dendritic segments), respectively, used for the analysis of dendritic complexity.

After decapitation, the unfixed brains were impregnated in the Golgi solution for 14 days and embedded in celloidin. One-hundred and fifty micrometre tissue sections were prepared and developed by using a modified Golgi–Cox technique (for methodological details, see Bock et al., 2016). For each hemisphere two pyramidal neurons located in layer II/III of the ventromedial prefrontal cortex (vmPFC) comprising the infralimbic (IL) and prelimbic (PL) cortex were analyzed (Figure 1A). The brain region was defined according to the rat brain atlas (Paxinos and Watson, 2004) and the degu brain atlas (Kumazawa-Manita et al., 2018). All neurons were reconstructed at a final magnification of 1,000× using a computer-based neuron tracing system (NEUROLUCIDA^®^, MicroBright-Field, Williston, VT, USA), which allows the quantitative three-dimensional analysis of complete dendritic trees. For each animal two neurons per hemisphere, that is four neurons per animal, were analyzed. Neurons selected for analysis (representative example in Figure 1B) had to fulfill the following criteria: (1) localization within the boundaries of the vmPFC, (2) uniform and complete staining of apical and basal dendritic trees within the 150 μm section, (3) apical dendrites had to branch regularly into a series of bifurcating branches divided into primary, secondary, tertiary, etc. and (4) sufficient distance from neighboring neurons, glia or blood vessels, which could obscure their morphology. All protrusions, thin, stubby, or mushroom type were counted as spines if they were in direct continuity with the dendritic shaft. No attempt was made to correct for hidden spines (Feldman and Peters, 1979), as the use of visible spine counts for comparison between different experimental conditions had been validated previously (Horner and Arbuthnott, 1991). The following parameters for each reconstructed neuron were quantified: (i) dendritic length in μm; (ii) spine frequency representing the number of visible spines per 10 μm dendritic length; (iii) number of visible spines; and (iv) dendritic complexity. All protrusions, thin, stubby, or mushroom type were counted as spines if they were in direct continuity with the dendritic shaft. The length of the dendritic trees was measured by tracing the entire dendrite in three dimensions while counting dendritic spines. Spine frequency was calculated separately for the apical and basal dendrites of each neuron. For a more detailed analysis, three parallel analysis strategies were performed: (1) To assess whether spine changes are confined to specific areas of the dendritic field, the natural branches (segments) of the apical and basal dendritic trees were numbered consecutively (primary, secondary, tertiary, etc.) from proximal to distal (Bock and Braun, 1999a,b; Bock et al., 2016; Figure 1C). Spine frequency was calculated: (i) as the average for the complete apical or basal dendrite; and (ii) as the average for the individual branching orders (see above). (2) Since it turned out that the most distal dendritic segments showed the most pronounced differences between the rearing groups we applied an additional analysis: the values for each parameter (dendritic length, spine frequency, spine number) were pooled for the 3rd-5th apical branch order or the 2nd–4th basal branch order, respectively. (3) To obtain more detailed information about changes of dendritic complexity, a three-dimensional version of the Sholl analysis (Sholl, 1953) was performed, in which concentric spheres at 50 μm intervals were placed around the soma and the number of intersections between dendrite and Sholl sphere was calculated (Figure 1D). All analyses were conducted by an experimenter who was unaware of the experimental conditions of the animals.

### Statistical Analysis

A two-way ANOVA was performed to test for potential differences within the main factors rearing condition (+F, −F, MA) and sex (male, female) and potential interaction of these factors. For a more detailed comparison of the individual experimental groups, a one-way ANOVA for male and female groups was conducted and in case of significance (*p* ≤ 0.05) a Student-Newman-Keuls-(SNK-) test *post hoc* tests was applied. The data for each hemisphere were pooled and analyzed separately, resulting in two values per animal. As described above we compared values of dendritic length, spine frequency, and spine number for: (i) complete neurons; and (ii) 3rd–5th order segments for apical dendrites, 2nd–4th order segments for basal dendrites. Also, values for comparison of dendritic complexity were derived from Sholl analysis.

## Results

In the present study, we aimed to analyze if growing up without a male caregiver (single mother family, −F group) may affect neuronal morphology in the vmPFC of periadolescent degu pups when compared to pups from a male and female biparental control family (control group). Moreover, to analyze if differences between these two rearing conditions are specific for a male caregiver, we introduced an additional biparental group consisting of two female caregivers (mother/aunt family, MA group). Also, we wanted to test for potential differences between male and female offspring. Thus, we first applied a two-way ANOVA to test for differences between the three rearing conditions, between male and female animals and potential interactions between these main factors. Also, a one-way ANOVA with the subsequent *post hoc* test was applied separately for male and female offspring to get a more detailed comparison between the individual rearing conditions.

### Rearing-Induced Differences in Spine Frequency, Spine Number, Dendritic Length and Complexity

Two-way ANOVA for factors rearing condition and sex revealed several significant effects or strong tendencies for the factor rearing condition and interaction of rearing condition × sex, no significant effects were detected for factor sex (for details see Table 1).

**Table 1:** Results of two-way ANOVA for factors rearing, sex, and interaction of rearing condition × sex.

Dendrite	Parameter	Rearing condition	Sex	Rearing condition × sex
apical	spine frequency	0.3	0.67	0.75
	spine number	0.2	0.5	0.3
	dendritic length	0.2	0.2	0.3
	dendritic complexity	*0.074*	0.3	0.28
basal	spine frequency	*0.068*	0.4	0.3
	spine number	*0.058*	0.5	*0.056*
	dendritic length	*0.056*	0.8	*0.069*
	dendritic complexity	**0.05**	0.7	0.27
apical pooled values 3rd–5th segments	spine frequency	0.3	0.5	0.9
	spine number	**0.002**	0.7	0.89
	dendritic length	**0.008**	0.9	0.7
basal pooled values 2nd–4th segments	spine frequency	*0.091*	0.7	0.1
	spine number	**0.026**	0.5	0.2
	dendritic length	0.1	0.2	0.2

*Bold numbers with grey background indicate significant differences (*p* ≤ 0.05); numbers in italics indicate a trend towards significance (*p* < 0.1)*.

Since two-way ANOVA revealed some evidence for an interaction of rearing conditions × sex (basal spine number and basal dendritic length), we conducted a one-way ANOVA with an SNK posthoc test for males and females separately to test for differences between the individual experimental groups. This analysis revealed that rearing conditions affect neuromorphological parameters and that these effects differ in male and female offspring, as described in detail in the following section.

### Male Offspring

**Figure 2:**
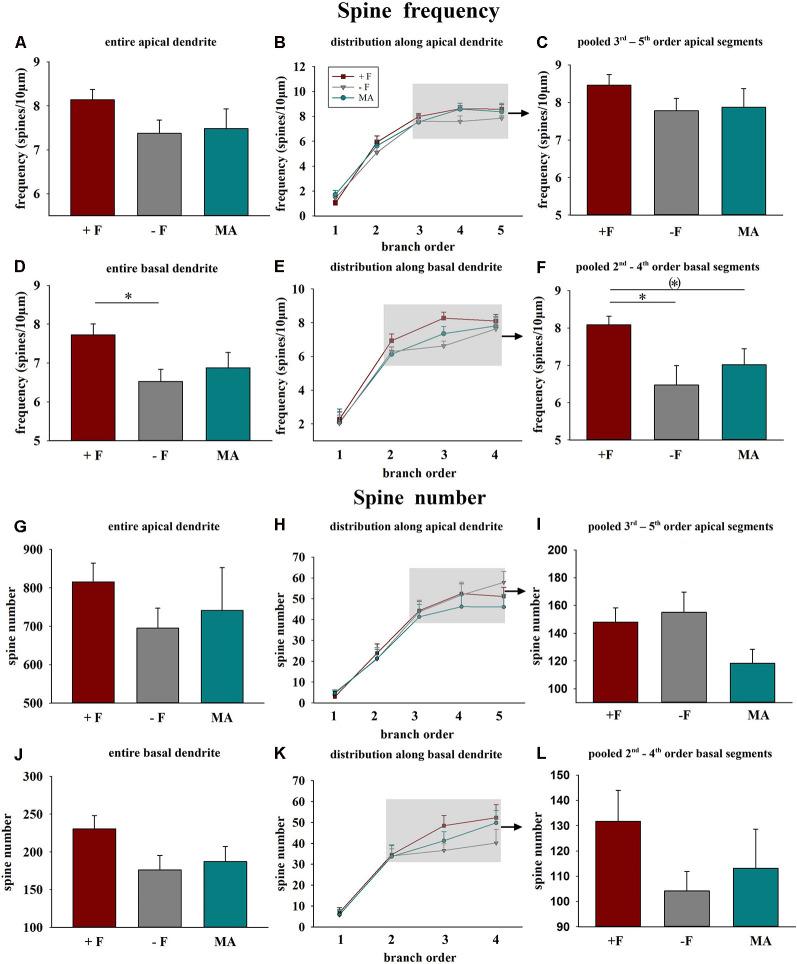
Effects of different rearing conditions on dendritic spine frequency and spine number in male degu offspring. **(A,D,G,J)** Mean dendritic spine frequency and spine number for entire apical **(A,G)** or basal dendrites **(D,J)**. **(B,E,H,K)** Distribution of spine frequency and spine number within the individual dendritic segments (branch orders) along apical and basal dendrites; rectangles in figures in the central column indicate the branch orders used for pooled segment analysis. **(C,F,I,L)** Mean spine frequency and spine number of pooled 3rd to 5th branch order segments in apical and the 2nd to 4th branch order segments in basal dendrites. +F, male and female biparental control group; −F, fatherless single mother group; MA, female biparental (mother and aunt) group. *p ≤ 0.05; ^(*)^*p* ≤ 0.1, SNK *post hoc* test.

*Spine frequency*: Analysis of vmPFC pyramidal neurons using a one-way ANOVA indicated significant differences in the average spine frequency for the complete basal dendrites between the three treatment groups (*p* = 0.035). *Post hoc test* revealed a significantly lower spine frequency of offspring from the single mother (−F) group compared to offspring from the biparental (+F) group (*p* = 0.034, 16% reduced, Figure 2D). Similarly, analysis for the pooled values of the 2nd to 4th basal dendritic segments indicated a significant difference between the treatment groups (*p* = 0.019) and the *post hoc* test revealed a significantly lower spine frequency for the −F group compared to the +F group (*p* = 0.017, 20% decrease, Figures 2E,F) in this basal segments. Also, the *post hoc* test showed a tendency towards a lower basal spine frequency in the mother-aunt (MA) group compared to the offspring of the +F group (*p* = 0.062, 13% decrease, Figures 2E,F). For the apical dendrites, no significant effects in spine frequency were found between the experimental groups (Figures 2A–C). Also, no differences neither for spine number (Figures 2G–L), dendritic length nor for dendritic complexity were observed in male animals.

### Female Offspring

**Figure 3:**
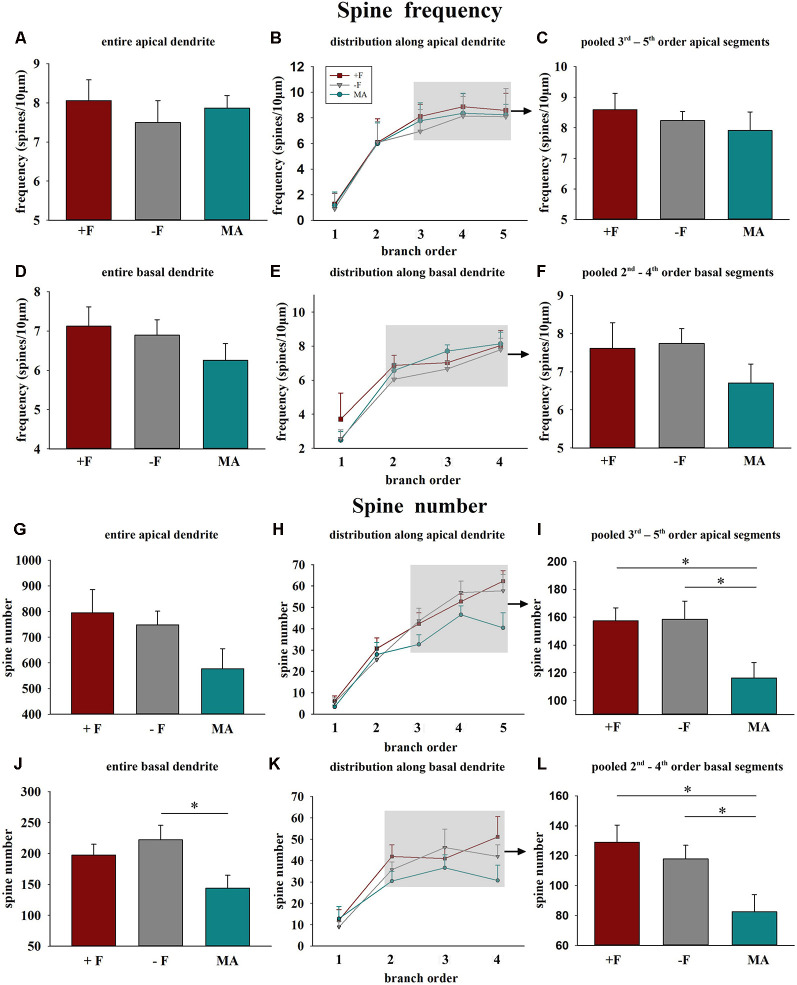
Effects of different rearing conditions on dendritic spine frequency and spine number in female degu offspring. Diagrams in the left column illustrate mean dendritic spine frequency and spine number for entire apical **(A,G)** or basal dendrites **(D,J)**. The diagrams in the center column illustrate the distribution of spine frequency **(B,E)** and spine number **(H,K)** along the individual dendritic segments (branch orders) of apical and basal dendrites. The grey shaded rectangles indicate the branch orders that were used for the pooled segment analysis (for details see “Materials and Methods” section). Diagrams in the right column illustrate the results of the pooled segment analysis, mean spine frequency, and spine number of the pooled 3rd to 5th branch order segments in apical **(C,I)** and the 2nd to 4th branch order segments in basal dendrites **(F,L)**. +F, male and female biparental control group; −F, fatherless single mother group; MA, female biparental (mother and aunt) group. *p ≤ 0.05, SNK *post hoc* test.

*Spine frequency*: No significant differences for spine frequency were found between the three rearing conditions neither on basal or on apical dendrites (Figures 3A–F).

**Figure 4:**
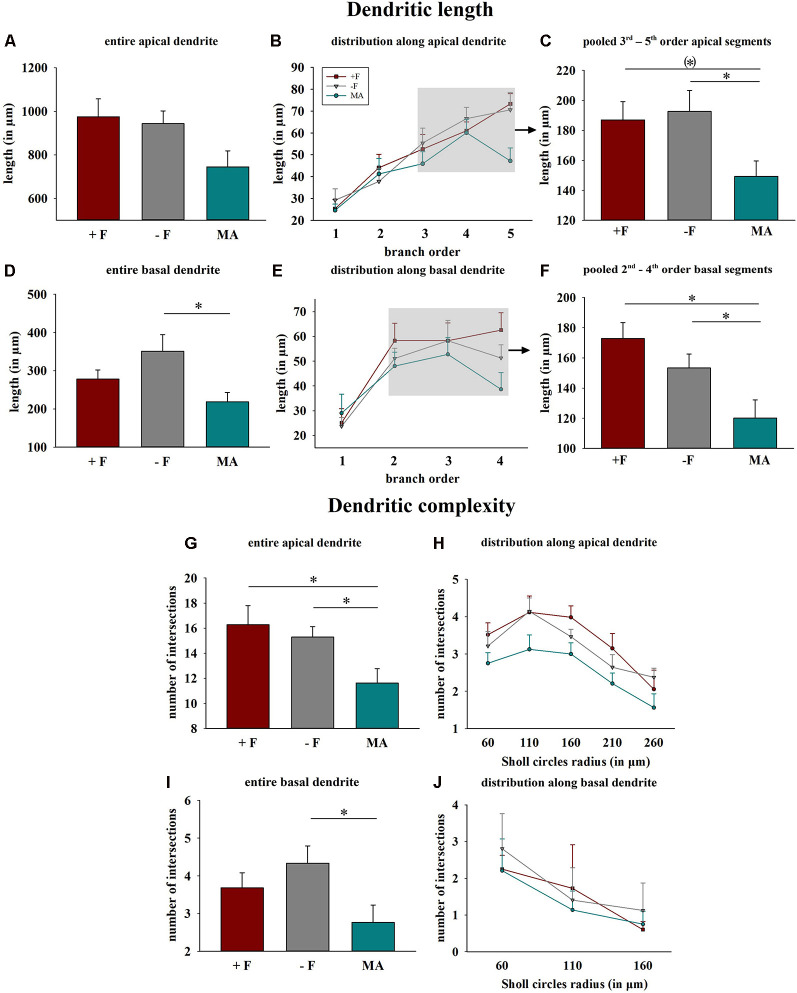
Effects of different rearing conditions on dendritic length and complexity in female degu offspring. **(A,D)** Dendritic length for entire apical **(A)** or basal **(D)** dendrites. **(B,E)** Distribution of mean dendritic length within the individual dendritic segments (branch orders) along apical and basal dendrites; rectangles in figures in the central column indicate the branch orders used for pooled segment analysis. **(C,F)** Dendritic length of the pooled 3rd to 5th branch order segments in apical and the 2nd to 4th branch order segments in basal dendrites. **(G,H,I,J)** Dendritic complexity indicated as intersections as a result of Sholl analysis; **(G,I)** sum across entire apical or basal dendrites, **(H,J)** distribution along apical and basal dendrites. +F, male and female biparental control group; −F, fatherless single mother group; MA, female biparental (mother and aunt) group. *p ≤ 0.05, ^(*)^*p* ≤ 0.1, SNK *post hoc* test.

*Spine number*: One way ANOVA in female offspring indicated significant differences in the average spine number for the complete basal dendrite between the three treatment groups (*p* = 0.037). *Post hoc* analysis revealed reduced spine number in female offspring of the MA group compared to −F offspring (*p* = 0.031, 35% decrease, Figure 3J). Similarly, analysis for the pooled values of the 2nd to 4th basal dendritic segments indicated a significant difference between the treatment groups (*p* = 0.012) and the *post hoc* test revealed a significantly lower spine number for the MA group compared to the +F group (*p* = 0.015, 36% decrease) as well as for the −F group (*p* = 0.019, 30% decrease, Figures 3K,L). While no significant effects were observed for the complete apical dendrite (Figure 4A), significant differences between the experimental groups were also observed for the pooled values of 3rd to 5th dendritic segments of apical dendrites (*p* = 0.025) and the *post hoc* test revealed a significantly lower number in female animals of the MA group compared to female animals of the +F group (*p* = 0.026, 26% decrease, Figure 3G) as well as of the −F group (*p* = 0.035, 27% decrease, Figures 3H,I).

*Dendritic length and complexity*: One way ANOVA for total basal dendritic length indicated significant differences between the three experimental groups (*p* = 0.027), and the *post hoc* test revealed reduced dendritic length in offspring from the MA group compared to offspring from the −F group (*p* = 0.021, 38% reduction, Figure 4D). Also, analysis for the pooled values of the 2nd to 4th basal dendritic segments indicated a significant difference between the treatment groups (*p* = 0.006), and the *post hoc* test revealed for this basal segments reduced dendritic length in the MA group compared to the +F group (*p* = 0.006, 30% reduction) as well as to the −F group (*p* = 0.028, 22% reduction, Figures 4E,F). While no significant effects were observed for the entire apical dendrites, one way ANOVA indicated differences for the pooled 3rd to 5th dendritic segments of apical dendrites between the experimental groups (*p* = 0.04) and the *post hoc* test revealed significantly reduced length of these apical segments in females from the MA group compared to females from −F group (*p* = 0.044, 22% reduction) as well as a strong tendency compared to the −F group (*p* = 0.053, 20% reduction, Figures 4B,C).

**Figure 5:**
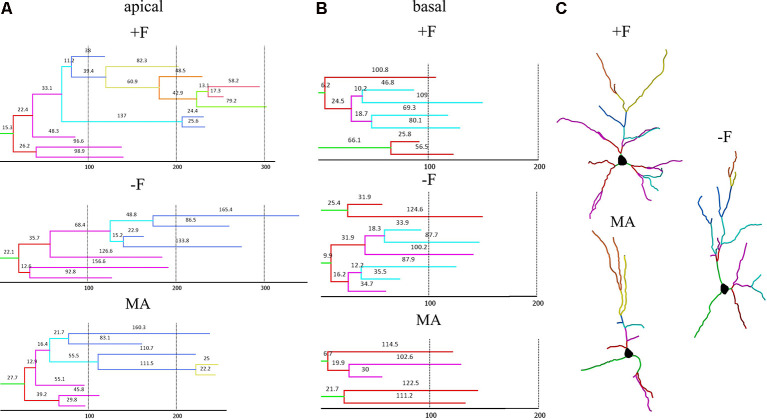
Dendrograms and reconstructions of representative neurons in the different rearing groups to visualize the differences in dendritic length and complexity in female offspring. **(A,B)** Representative dendrograms for apical and basal dendrites; hatched vertical lines and numbers on each segment indicate length in μm. **(C)** Reconstructions of representative pyramidal neurons; branch orders are labeled by individual colors. +F = biparental control group, −F, fatherless group reared by mother; MA, reared by mother and aunt.

For dendritic complexity as analyzed with Sholl analysis ANOVA indicated a difference between the experimental groups in the basal dendrite (*p* = 0.05) and *post hoc* analysis revealed reduced basal dendritic complexity in the MA female group compared to the −F female group (*p* = 0.039, 36% reduction, Figures 4I,J). Also, for dendritic complexity of apical dendrites, ANOVA indicated a difference between the female experimental groups (*p* = 0.018) and the *post hoc* test revealed a reduction apical dendritic complexity in the MA group compared to the +F group (*p* = 0.024, 28% reduction) as well as to the −F group (*p* = 0.023, 24% reduction, Figures 4G,H). Results for dendritic complexity are illustrated as representative dendrograms and reconstructions of representative pyramidal neurons in Figure 5 (dendrograms Figures 5A,B; representative reconstructions Figure 5C).

## Discussion

The target area of the present study, the vmPFC, is implicated in various behavioral aspects and specifically related to the control of socio-emotional behavior, behavioral flexibility, and cognitive functions. Although the homology of vmPFC subregions between humans and rodents is still under debate, it is important to note that the vmPFC is a key modulatory area of specific cortical and subcortical networks that include the amygdala, hippocampus, bed nucleus of stria terminalis, periaqueductal grey, anterior cingulate cortex and nucleus accumbens (Ko, 2017; Hiser and Koenigs, 2018). Previous studies in *Octodon degus* revealed that growing up in fatherless family results in various alterations of neuronal networks, which are indicative of a shift in homeostatic synaptic plasticity, i.e., the balance of excitatory and inhibitory activity in the prefrontal cortex. This view is supported by findings demonstrating that reduced excitatory input onto neurons in the PFC (reflected by decreased spine density and/or number) is paralleled by an increase in the density of different subtypes of inhibitory interneurons in the OFC as well as in the vmPFC (PL/IL; Braun et al., 2011). Although it is premature, without behavioral correlates indicative for prefrontal dysfunction, to offer a functional interpretation for the reduced synaptic density in father-deprived male offspring, it is tempting to speculate that these neuroanatomical changes in response to paternal deprivation might indicate that prefrontal regions may become hypofunctional. In support for this interpretation, we observed in degu pups, which were repeatedly separated from their parents and family during the first 3 weeks of life, that they display decreased metabolic brain activity in several brain areas including prefrontal cortical areas such as ACd, OFC as well as the PL and IL (Bock et al., 2017). This finding matches observations of functional imaging studies, which revealed that children suffering from early neglect and emotional deprivation display prefrontal hypofunction (Chugani et al., 2001).

### Paternal Care Influences Dendritic and Synaptic Development

So far, only a few studies compared the effect of paternal care on the brain and behavioral development in male and female offspring. Although on the “mathematical level” the two-way ANOVA revealed no clear significant effects for the factor sex, a trend for interaction between rearing conditions and sex was detected. The comparison of individual groups—reflecting the “biological” view—revealed that paternal care affects dendrites and spine synapses in the vmPFC differently in male and female offspring. While in paternally deprived male offspring spine frequency on basal dendrites was reduced without changes in dendritic length and complexity, this effect was completely absent in paternally deprived females. In contrast, reductions of spine number and dendritic length and complexity in the mother/aunt group were restricted to female offspring, whereas male offspring appeared not to be affected by this rearing condition (see below). Sex-specific consequences of paternal deprivation have also been observed in other species, where either males or females were more severely affected. Moreover, the impact of paternal deprivation on brain development of his offspring is not confined to degus but has also been observed in other biparental rodents and non-human primates, which suggests that similar neuronal changes may occur in the human brain in response to paternal deprivation. For instance, electrophysiological studies in the mPFC of California mice observed lower firing rates of low-spiking pyramidal neurons in father-deprived females compared to biparentally raised females, whereas no significant differences in basal firing rates of both low-spiking and high-spiking neurons were seen in father-deprived and biparentally raised males (Bambico et al., 2015). Paternal deprivation-induced neuronal changes have also been reported in limbic brain regions, which are connected to the mPFC, including the hippocampal formation. In father-deprived prairie voles (Microtus ochrogaster) glucocorticoid receptor *β* (GR*β*) protein expression was elevated in the hippocampus of females, increased corticotropin-releasing hormone receptor 2 (CRHR2) protein expression was observed in the hippocampus of males, while in both sexes paternal deprivation resulted in decreased hippocampal CRHR2 mRNA (Tabbaa et al., 2017). A recent study in the biparental mandarin vole demonstrated that pre-weaning paternal deprivation at postnatal days 14–21 reduced neurogenesis in the dentate gyrus, predominantly in female offspring (Wu et al., 2014), and only female offspring showed decreased expression of Brain-Derived Neurotrophic Factor (BDNF) and the glucocorticoid receptor (NR3C1; Wu et al., 2014). In contrast, decreased dendritic spine density was observed in the dentate gyrus of both males and females (He et al., 2018).

### A Female Caregiver Cannot “Buffer” the Neuronal Consequences of Paternal Deprivation

Most experimental studies in bi-parental non-human species apply paternal deprivation paradigms, where the father is removed from the social unit. These studies demonstrate that the absence of the father affects various behavioral traits in the progeny, including anxiety, aggression, social behavior and response to reward (Bester-Meredith and Marler, 2003; Frazier et al., 2006; McGraw and Young, 2010; Wang et al., 2012; Yu et al., 2012; Birnie et al., 2013). When interpreting findings from paternal deprivation studies in animal models it has, however, to be kept in mind that the observed consequences on the brain and behavioral development might not be specific to the absence of a male caregiver but might rather be due to single-parent rearing conditions. This question was experimentally addressed in our study by replacing the father with another female caregiver (the sister of the dam, i.e., the “aunt”) resulting in a female biparental family. Our results revealed that a female caregiver cannot protect from or compensate for the neuronal changes induced by growing-up in a fatherless environment, but rather induces additional neuronal changes, particularly in the vmPFC of female offspring. Again, it is tempting to speculate that these changes might be indicative of prefrontal dysfunction. However, the socio-emotional environment provided by a female biparental family remains to be investigated in more detail, to assess whether it represents an “impoverished” or an overprotective or stressful environment.

## Data Availability Statement

The datasets generated for this study are available on request to the corresponding author.

## Ethics Statement

The animal study was reviewed and approved by Ethics committee of Saxony-Anhalt, Germany.

## Author Contributions

TS conducted 3D analysis of stained neurons, statistical analysis, preparation of figures and was involved in writing parts of the mansucript. JB did brain preparations, guided histological staining, 3D analysis and statistics and wrote signifcant parts of the manuscript. KB led the project, planned the experimental design, guided analysis including statistics, wrote signficant parts of the manuscript and did final editing of the submitted draft.

## Conflict of Interest

The authors declare that the research was conducted in the absence of any commercial or financial relationships that could be construed as a potential conflict of interest.
